# Active Medical Learner Engagement Results in the Discovery That One Size Does Not Fit All in Overcoming COVID-19 Vaccine Hesitancy

**DOI:** 10.3390/vaccines11071140

**Published:** 2023-06-24

**Authors:** Arash Salari, Manpreet K. Singh, Shuja Ayouby, Sanmisola George, Kimngan Nguyen, Guillermo Daniel Peverini, Nicolette Lam, Timothy Allison-Aipa, Susanna Zamarripa, Shunling Tsang, Anthony Firek

**Affiliations:** 1 Department of Family Medicine, Riverside University Health System, Moreno Valley, CA 92555, USA; 2 Riverside School of Medicine, University of California, Riverside, CA 92507, USA; 3 Comparative Effectiveness and Clinical Outcomes Research Center, Riverside University Health System, Moreno Valley, CA 92555, USAa.firek@ruhealth.org (A.F.)

**Keywords:** COVID-19, vaccine hesitancy, race, determinants, SARS-CoV-2, vaccine acceptance, patients

## Abstract

Vaccine hesitancy is an ongoing public health concern defined as the refusal of a vaccine that is readily available. Therefore, we developed a project to explore why patients in a safety net medical center were hesitant or refused the COVID-19 vaccine. The project was conducted by healthcare learners to promote “learning by doing”. Responses were collected through a previously developed and ongoing survey among both hospitalized and ambulatory patients that had no previous history of COVID-19 infection, were currently infected, or had recovered from COVID-19. Results were analyzed using a priori power analysis and Chi-squared test. We discovered that different self-reported ethnic groups had different reasons for vaccine hesitancy; specifically, 69% of Black/African American respondents stated that their main reason for hesitancy was vaccine safety compared to 13.9% of non-Hispanic Whites (*p* = 0.005). Furthermore, our cohort was significantly more likely to disagree rather than agree with the statement: “getting vaccinated is important for the health of others in my community”(*p* = 0.016). The learners discovered that a more specific approach to vaccine education would be required to understand and overcome vaccine hesitancy in our cohort of socioeconomic and ethnically diverse groups.

## 1. Introduction

The COVID-19 pandemic that began in Wuhan, China, in 2019 continues worldwide and has now expanded largely due to the omicron variant of SARS-CoV-2. As of 20 June 2022, there have been over 539 million confirmed cases and over 6 million deaths globally. Specifically, the United States has had over 1 million deaths due to COVID-19 [1].

Although the COVID-19 vaccine may not be 100% effective in preventing infection, it helps prevent severe complications and death. Unvaccinated individuals are at risk for hospitalization and increased mortality. The study by Tenforde et al. reported that up to 84.2% of those who are hospitalized due to COVID-19 and have disease progression are unvaccinated [2]. Therefore, this illustrates that high community vaccination rates are vital for creating a level of community immunity that lowers the number of SARS-CoV-2 cases and deaths.

Among a number of determinants, a successful vaccine program requires that susceptible subjects have confidence in the efficacy and safety of the vaccine itself. A crucial element is the degree of vaccine hesitancy which can lead to lower vaccination rates and increased adverse health outcomes. Understanding how a local population that is served by a medical center views the risk and benefits of the vaccine and how medical enterprises address these issues is the basis for improving the efficacy of vaccine uptake. Although the global acceptance of vaccination is quite strong, certain populations remain skeptical. Vaccine hesitancy is defined as the “delay in acceptance or refusal of vaccines despite availability of vaccination services” [3]. Hesitancy can stem from a multitude of reasons and can change over time. Some examples include the fear of side effects, distrust in the government, and inaccessible vaccine clinics [4].

Even in the face of emerging evidence supporting vaccine benefits, hesitancy continues to impede optimal vaccination rates within the population. The literature regarding vaccine hesitancy among minority groups has been mixed. Some studies reveal the highest level of hesitancy among those who identify as Black, while other studies demonstrate that Blacks showed less hesitancy for the COVID-19 vaccine compared to non-Hispanic whites (NHW) [5,6,7]. Understanding both these subject-specific issues with vaccination and the medical enterprise challenges in delivering vaccines is critical to success.

A project completed by Doherty et al. in underserved communities in North Carolina surveyed 948 participants and found the prevalence of vaccine hesitancy to be 74% among their Black participants, 62.7% among their NHW participants, and 59.5% among their Latinx participants [5]. Another project completed by Famuyiro et al. assessed vaccine readiness among healthcare workers at three university and community-based health centers [6]. Famuyiro et al. discovered that even after adjusting for age, sex, and perceived risk, non-Hispanic Blacks were significantly less likely to agree to vaccines [6]. On the other hand, van den Broek-Altenburg et al. had opposing results when surveying the four largest states (New York, California, Texas, and Florida) [7]. The author found that vaccine hesitancy was highest among their white-identifying participants and lowest in their Black participants, with a total of 28% of their participants refusing to receive the vaccine [7].

Underserved and low-socioeconomic communities have been disproportionally affected by COVID-19 [8]. For example, in Chicago, African Americans make up 50% of COVID-19-related deaths, although they only represent 33% of the city’s population [9]. Current literature regarding vaccine hesitancy in underserved and low socioeconomic populations is largely unknown. Therefore, we developed a project to discover why decisions were made not to vaccinate within our population. The project was innovative as it was conceived, designed, and conducted by engagement of healthcare learners to provide a “hands-on” educational experience and provide critical clinical information.

Our medical center, Riverside University Health System (RUHS), is the primary safety net hospital for Riverside County, California. RUHS is an integrated health network in Riverside County, California that includes a 439-bed county Medical Center, 13 federally qualified health centers, primary and specialty clinics, and departments of Behavioral and Public Health. As the 10th largest county in the nation and with a Healthy Places Index (HPI) score in the 2nd lower quartile in the state of California, RUHS serves a highly diverse population with a significant level of health disparity and is considered at high risk for COVID-19-related complications [10].

At the time of the release of the vaccines, we initiated a medical center-wide project to improve the quality and processes for the delivery of vaccines. Our initial project was based on the development of a comprehensive 69-question survey evaluating determinants of vaccine hesitancy within our employee population [11].

Based on the previous projects, we extended the survey to patients who were not yet vaccinated. At the time of the survey, many of our medical residents had been involved in COVID-19 response efforts and vaccination campaigns. Ongoing discussions between students, medical residents, and faculty at RUHS regarding vaccine hesitancy led to the development of this project as a way to obtain direct input from patients we serve regarding attitudes and beliefs about COVID-19 vaccines. This project provided an educational experience for medical learners through hands-on surveying of patients from both hospitalized and ambulatory settings who have not received the COVID-19 vaccine and do not plan to receive the vaccine in the future.

It is critical to understand why individuals decide to remain unvaccinated as this will inform our local healthcare teams on effective patient-centered interventions to increase vaccination rates. This paper offers survey results after the approval of Pfizer, Moderna, and Johnson & Johnson vaccines in the United States. Vaccine uptake is particularly challenging in our unique patient population due to the variable and overall low health literacy. The overall project aims to utilize medical residents, nursing, and university students to provide the opportunity to learn by interacting with the unvaccinated patient population in our safety net hospital using our current survey and discover the factors leading to their vaccine hesitance. We anticipate the responses obtained from this robust survey will improve our local processes to enhance vaccine success and create targeted resources for our patient population.

## 2. Materials and Methods

**Figure 1:**
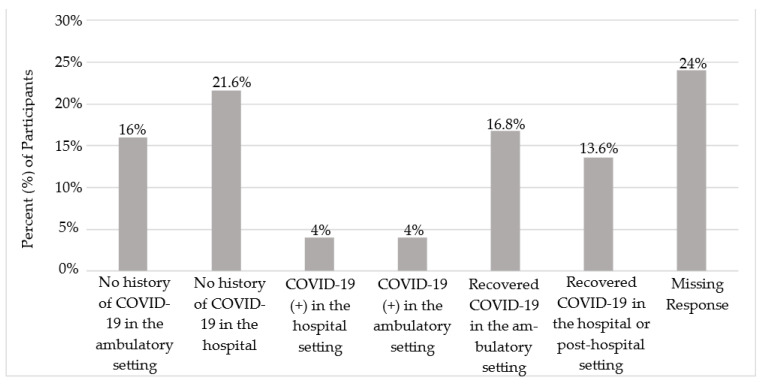
Bar graph depicting the percent of participants with no history of COVID-19 infection in the ambulatory or hospital setting and with COVID-19 infection in the ambulatory or hospital setting.

The project team was developed and led by a senior Family Medicine resident (AS) and assisted by medical residents, nursing professionals, medical students, and undergraduate university students. The two guiding objectives for this education quality improvement project were to engage medical learners in the opportunity to interact with our local patient population and to enhance their educational experience in understanding the barriers to successful vaccination. The second objective was to provide our local clinical care quality improvement team with information that may improve vaccine access and uptake within our center. Oversight was provided by a senior clinical investigator (AF) and faculty advisor (ST). The medical learner team utilized our previously developed internal validated survey administered to employees of RUHS [11]. Patients were approached and verbally consented per our standard medical center policy for quality improvement projects. Anonymity was provided as all responses were collected in a de-identified fashion. Survey responses were collected among both hospitalized and ambulatory non-vaccinated patients that either had no history of COVID-19 infection, were currently infected, or had recovered [Figure 1]. The project collected surveys between August 2021 through November 2021 at Riverside University Health System (RUHS medical center and Moreno Valley Community Health Center, the main teaching clinic for the Family Medicine Residency Program). Patients included in the survey were under the care of the family medicine or internal medicine team at RUHS. In addition, to be eligible, patients had to not be vaccinated at the time of the survey and not have plans to receive the COVID-19 vaccine in the future.

Vaccine hesitancy is defined as the refusal of a vaccine that is readily available. Therefore, when conducting our survey, vaccine hesitancy was defined as patients who responded with “no’’ when asked if they were currently vaccinated and responded “no” if they were willing to get vaccinated at a future date or time in the inpatient or outpatient setting.

Healthcare learners, specifically family medicine residents and medical students, conducted the surveys. The healthcare learners were instructed to read the survey as written to the participant to reduce variability among different individuals. In addition, healthcare learners were instructed to refrain from speaking about personal questions or attempting to influence the participant’s stance on the COVID-19 vaccine. Healthcare learners were only allowed to answer questions regarding instructions on how to answer various question types, such as ranking versus multiple choice. Patients were also given the option to complete the survey on paper or on a tablet device when available. In addition, to include patients who tested positive for COVID-19, patients testing positive had the opportunity to fill out the survey over the phone. These various modalities were used to make the survey more accessible to various levels of technological proficiency.

Some questions were designed to be ranking questions. Participants were asked to rank predetermined responses from one to six. From the responses, the main reason for vaccine refusal was what the participants ranked as number 1 on their list as the top reason. The main question asked was, “please list your major reasons for your choice of deciding against COVID-19 vaccinations even though others are telling you to get vaccinated”. Responses included were “I do not believe vaccines are safe”, “I am healthy and have a low risk of getting COVID-19”, “I don’t believe the COVID-19 vaccine is very effective”, “I believe you can get COVID-19 from the vaccine”, “I have an allergic reaction to the vaccine”, and “mandating vaccines are against my human rights/personal freedom”.

The survey also addressed factors that would possibly influence the participant’s decision to receive the vaccine, such as financial compensation, vaccine safety confirmation, job requirements, and ease of access. The goal of these questions was to determine if external incentives would influence vaccine hesitancy and thus motivate participants to change their minds about the COVID-19 vaccine.

All variables, including age, race, gender, and socioeconomic status, were self-reported. A priori power analysis was carried out in IBM SPSS version 27. Demographic information was analyzed using descriptive statistics, such as means and frequencies, while group comparisons were made using chi-square tests.

## 3. Results

All the engaged healthcare learners reported a highly positive experience with new insights into how people determine health-related choices. There were 125 patients that completed the survey, with 25 self-identifying as Black/African American, 42 as Hispanic or Latino, and 42 as non-Hispanic White. A total of 65 participants identified as female, and 50 participants identified as male. There were 25 participants between the ages of 18–29 years old, 31 participants between 30–39 years old, 16 participants between 40–49 years old, 17 between 50–59 years old, and 26 participants over the age of 60 [Table 1].

Participants were from various socioeconomic groups [Table 2]. Twenty-nine participants reported their income was less than USD 20,000 annually, while 37 reported their income was between USD 20,000–49,999. Twenty-two participants reported that their income was between USD 50,000–89,999 annually, while seven participants reported their income was between USD 90,000–119,000. Lastly, four participants reported that their annual income was greater than USD 120,000.

Based on the survey results, unvaccinated patients were significantly more likely to disagree with the statement: “Getting the vaccine is a good way to protect me from disease”: agree (*n* = 2; 9.5%) vs. disagree (*n* = 48; 53.3%), *p* < 0.001. When the data were analyzed, 69% of Black/African American respondents stated that the main reason for vaccine hesitancy was a concern about vaccine safety, compared to 13.9% of non-Hispanic Whites (*p* = 0.005). In addition, 40% of divorced patients (*n* = 12) rated being “healthy and low risk of getting COVID-19” as the least important factor contributing to their vaccine hesitancy compared to 0% of single patients (*n* = 48) (*p* = 0.013).

When asked about their stance on various statements in our survey, unvaccinated patients were significantly more likely to disagree with the statement: “vaccines are important for my health”: agree (*n* = 3; 14.3%) vs. disagree (*n* = 46; 50.0%), *p* = 0.003. Unvaccinated patients were significantly more likely to disagree with the statement: “getting the vaccine is important to protect the health of my family”: agree (*n* = 3; 14.3%) vs. disagree (*n* = 51; 55.4%), *p* < 0.001. Unvaccinated patients were significantly more likely to disagree with the statement: “getting the vaccine is a good way to protect myself from disease”: agree (*n* = 2; 9.5%) vs. disagree (*n* = 48; 53.3%), *p* < 0.001. Unvaccinated patients were significantly more likely to disagree with the statement: “getting vaccinated is important for the health of others in my community”: agree (*n* = 2; 9.5%) vs. disagree (*n* = 35; 38.0%), *p* = 0.016.

When asked about factors that could influence their decision about receiving the vaccine, such as getting paid time off work, 72.8% (*n* = 91) said they either strongly disagreed or disagreed. 55% (*n* = 69) said they would not consider the vaccine even if it was a requirement for their job. In addition, when asked if receiving a financial incentive would influence their decision to receive the vaccine, 72% (*n* = 90) of participants strongly disagreed or disagreed with the statement. Interestingly, 72.8% (*n* = 91) of participants also strongly disagreed or disagreed with the statement regarding the positive impact of vaccine promotion in social media networks. On the other hand, 56.8% (*n* = 71) said they would consider getting the vaccine if they were sure that the vaccine was safe, and 54.4% (*n* = 68) said they would consider receiving the vaccine if they were confident it was effective and those who received the vaccine did not get COVID-19.

## 4. Discussion

The engagement of the healthcare learners in the organization, design, and collection of the responses and the ability to interact directly with the participants resulted in a highly positive experience. Understanding these operational challenges in developing a local quality improvement project within our diverse population and our specific medical center was one of the objectives. A second project is in the development phase to address the specific educational experience of the team members. Based on their interactions and the data collection, the project revealed personal beliefs, self-reported race, and marital status as factors that influenced vaccine hesitancy. It is important to note that our unvaccinated patients disagreed with statements regarding vaccines and their relationship to one’s personal and family health. Therefore, promoting the vaccine as a way to obtain herd immunity and protect one’s community may not appeal to the unvaccinated population in our cohort. How this finding may translate into other centers is unknown, but it supports the need to conduct projects such as this at the local level.

The responses provided insight for local process improvement teams. We learned that 19.2% of the participants’ main concern was vaccine safety, while 16% of participants were concerned about the effectiveness of the vaccine [Table 3]. The phrasing of pro-vaccine advertising needs to be adjusted to target those who are hesitant with a potentially alternate focus. Chou and Budenz 2020 emphasized the importance of emotion in communication regarding the vaccine, and anti-vaccine campaigns targeted fear and anxiety already present in the community [12]. Further communication efforts need to focus on the positive outcomes of the vaccine, such as fewer hospitalizations, increased survival rates, and safety profiles. Therefore, this illustrates the potential benefit of encouraging healthcare workers and residents to address misconceptions and generalized fear regarding the vaccine. Future studies can delve into the impact of interventions that directly relate to vaccine safety and efficacy on vaccine uptake.

In addition, our project revealed that vaccine mandates for work are not effective in changing the minds of those who are not vaccinated. In addition, our cohort revealed that 72.8% of participants did not believe that the promotion of the COVID-19 vaccine would affect their decision to receive the vaccine. This reveals that for this cohort, strong-rooted beliefs cannot be adequately tackled through force via institutional guidelines or excessive advertising. Rather, more efforts need to be put into targeted outreach that gives individuals the ability to communicate. We propose the implementation of focus groups to provide hesitant community members with a safe space to express their concerns. Furthermore, informed physicians and/or other trusted providers, such as pharmacists, can address any misconceptions and fears regarding the vaccine [13]. Through this mechanism, we can focus on the positive safety profile and effectiveness of the vaccines without fear or judgment about vaccine hesitancy.

Our project revealed there were differences in the reasons for hesitancy based on self-reported ethnicity, as 69% of Black/African American respondents stated that the main reason for vaccine hesitancy was concern about vaccine safety. Therefore, future studies can remeasure participants’ vaccine hesitancy after receiving information focused on vaccine safety. Currently, at our safety-net hospital system, we aim to provide patients with information regarding the risks of COVID-19 itself, including long COVID-19 and post-COVID-19 conditions on their after-visit summaries so that they fully understand the risks of refusing vaccination after their visit and on their own time. If patients have questions after reading the summary, they are encouraged to contact their provider for clarification on any of the information provided.

The guiding medical center’s objective of this project is to reduce morbidity and mortality from COVID-19 for all our patients. With every COVID-19 wave, the hospital census increases, elective cases are impacted, and create overall strain on our healthcare system. Vaccines remain the best line of defense against COVID-19 [14].

A limitation of our project is that patient participation was not obtained in those who were non-verbal due to intubation or a comatose state. In addition, many eligible patients did not participate in the survey. A patient’s refusal to participate may stem from the belief that the interviewer would inherently try to change their mind and/or make judgments about their reasoning. However, we believe that our cohort, although small, provides us with insight into factors we can target directly at our medical center. Although this may not address everyone’s concerns and reasons, it is a step in the right direction.

Follow-up studies would benefit from more open-ended question responses. Our survey questions had multiple pre-written responses for the participant to choose from. Providing the patient with open-ended questions, especially regarding factors that may convince them to later receive the vaccine, could inform a health system on how to better provide information on COVID-19 vaccines to those who are hesitant.

We did not include patients that have made a decision to be vaccinated in the future. The project was focused on those refusing vaccination or strongly uncommitted to vaccination. The reason for this distinction was for healthcare learners and our medical center administration to isolate those who had issues with the vaccine itself rather than the logistical obstacles that have prevented them from getting vaccinated. We acknowledge that the opinions of the participants were limited by the moment in time that they were collected, and the same participants may now have different views. Throughout the data collection, the number of surveys that were offered and declined was not counted to provide a more accurate representation of the number of our patients refusing the vaccine, as there were multiple surveyors. We attempted to minimize surveyor bias by using a scripted explanation of the objective of the survey and instructed surveyors to adhere completely to the words and instructions written on the surveys. In addition, all patients under the family medicine and internal medicine service were recruited to minimize variation in the types of patients between different surveyors. Selection bias may have affected our data, and those who declined the survey and were unvaccinated may have had a different response than those who filled out the survey. As all the responses were collected in a deidentified manner to protect patient confidentiality, we could not track or confirm follow-up vaccination uptake in those claiming they would be vaccinated in the future.

In any quality improvement project, the intent is to identify processes and procedures locally that can be then changed to effect improved outcomes for the patients and the medical enterprise. This includes providing the opportunity for our healthcare learners to engage in this process and promote the type of medical practice changes such as group interactions, patient-oriented engagement, literature review, and outcome discovery.

We also believe that our cohort of patients brings to light some of the reasons that individuals remain hesitant to receive the vaccine. We believe that this project was a great first step and provides insight into how vaccine education can be targeted in more hesitant populations.

## 5. Conclusions

In conclusion, our project using the “learn by doing” approach has achieved the stated objectives. Healthcare learners successfully organized and conducted the project, and overall, interviews with them indicated they felt it was beneficial, educational, and insightful. This approach could potentially improve their future practice habits, whether in nursing, medicine, pharmacy, or other allied health fields. The goal would be to have healthcare providers at all levels address misconceptions and fears regarding the vaccine. The responses our team obtained during the project revealed personal beliefs, race, and marital status as factors that influence COVID-19 vaccine hesitancy. In addition, our project demonstrated the importance of maintaining the power of individual choice and providing the tools to make the most educated choice for what is important for the patient. With the likelihood of new infections and viruses, understanding how to best advertise and address concerns from our community members is very important. With this insight generated locally, we hope our novel approach to information gathering and survey findings will result in increased vaccination rates and improved clinical outcomes.

## Figures and Tables

**Table 1:** Self-Reported Patient Demographics.

Variable	*n*_total_ = 125	%
**Race**		
Black/African American	25	20.0
Hispanic/Latino	42	33.6
Non-Hispanic White	42	33.6
Other	5	0.04
No Response	11	0.9
**Gender**		
Male	65	52.0
Female	50	40.0
No Response	10	0.8
**Age**		
18–29	25	20.0
30–39	31	24.8
40–49	16	12.8
50–59	17	13.6
60+	26	20.8
No Response	10	0.8

**Table 2:** Participants’ Socioeconomic Status.

Reported Annual Income	*n*_total_ = 125	%
<USD 20,000	29	23.2
USD 20,000–49,999	37	29.6
USD 50,000–89,999	22	17.6
USD 90,000–119,999	7	5.6
USD 120,000 or above	4	3.2
No Response	26	20.8

**Table 3:** Main reason for vaccine hesitancy.

Reason	*n*_total_ = 125	%
Mandating vaccines are against my human rights/personal freedom	26	20.8
I do not believe vaccines are safe	24	19.2
I do not believe the COVID-19 vaccine is very effective	20	16.0
I am healthy and have a low risk of getting COVID-19	14	11.2
I believe you can get COVID-19 from the vaccine	3	2.4
I have an allergic reaction to the vaccine	2	1.6
No Response	36	28.8

## Data Availability

The data presented in this study is available on request from the corresponding author.
